# Supplementary material for: A Review of Biomarkers and Their Clinical Impact in Resected Early-Stage Non-Small-Cell Lung Cancer
Source: Cancers (Basel). 2023 Sep 14;15(18):4561. doi: 10.3390/cancers15184561 (PMC10526902; doi:10.3390/cancers15184561)
Supplement: Supplementary file 1 [file cancers-15-04561-s001.zip › cancers-2546731-supplementary.pdf]

Supplementary Text S1. Detailed retrieval strategies in PubMed.

(((((((("Carcinoma, Non-Small-Cell Lung"[Mesh]) OR (Carcinoma, Non small cell lung[Title/Abstract])) OR (Lung carcinoma, Non-small-cell[Title/Abstract])) OR (Non small cell lung carcinoma[Title/Abstract])) OR (Non-small cell lung cancer[Title/Abstract])) OR ((("Adenocarcinoma of Lung"[Mesh]) OR (Lung adenocarcinoma[Title/Abstract])) OR (Adenocarcinoma, Lung[Title/Abstract]))) OR (Squamous cell lung cancer[Title/Abstract])) AND (((((("Surgical Procedures, Operative"[Mesh]) OR (Operative procedure[Title/Abstract])) OR (Surgical procedure[Title/Abstract])) OR (Operative surgical procedure[Title/Abstract])) OR ((((("Surgery" [Subheading]) OR (Operative therapy[Title/Abstract])) OR (Invasive procedures[Title/Abstract])) OR (Operative procedures[Title/Abstract])) OR (Operation[Title/Abstract]))) OR (Resection[Title/Abstract]))) AND ((((("Mutation"[Mesh]) OR (Mutations[Title/Abstract])) OR ((((((Programmed cell death-1[Title/Abstract]) OR (PD-1[Title/Abstract])) OR (Programmed cell death-ligand 1[Title/Abstract])) OR (PD-L1[Title/Abstract])) OR (Tumor mutational burden[Title/Abstract])) OR (TMB[Title/Abstract]))) OR (((("Neoplastic Cells, Circulating"[Mesh]) OR (Neoplasm Circulating Cells[Title/Abstract])) OR (Circulating Neoplastic Cells[Title/Abstract])) OR (Circulating Tumor Cells[Title/Abstract]))) OR (((((((("Biomarkers"[Mesh]) OR (Biological Markers[Title/Abstract])) OR (Biologic Markers[Title/Abstract])) OR (Immunologic Markers[Title/Abstract])) OR (Immune Markers[Title/Abstract])) OR (Serum Markers[Title/Abstract])) OR (Clinical Markers[Title/Abstract])) OR (Biomarker[Title/Abstract])) OR (Circulating Biomarkers[Title/Abstract])))).
